# Future of Telepresence Services in the Evolving Fog Computing Environment: A Survey on Research and Use Cases

**DOI:** 10.3390/s25113488

**Published:** 2025-05-31

**Authors:** Dang Van Thang, Artem Volkov, Ammar Muthanna, Andrey Koucheryavy, Abdelhamied A. Ateya, Dushantha Nalin K. Jayakody

**Affiliations:** 1 Department of Telecommunication Networks and Data Transmission, The Bonch-Bruevich Saint-Petersburg State University of Telecommunications, 193232 Saint Petersburg, Russia; thangdang.251196@gmail.com (D.V.T.); artemanv.work@gmail.com (A.V.); muthanna.asa@sut.ru (A.M.); akouch@mail.ru (A.K.); 2 EIAS Data Science Lab, College of Computer and Information Sciences, Prince Sultan University, Riyadh 11586, Saudi Arabia; aateya@psu.edu.sa; 3 Department of Electronics and Communications Engineering, Zagazig University, Zagazig 7120001, Egypt; 4 CIET/DEEE, Faculty of Engineering, Sri Lanka Institute of Information Technology, Colombo 10115, Sri Lanka; 5 COPELABS, Lusófona University, 1749-024 Lisbon, Portugal; 6 UNINOV-CTS & LASI, 2829-516 Caparica, Portugal

**Keywords:** 6G, cloud computing, fog computing, mobile communication, next-generation networking, telepresence

## Abstract

With the continuing development of technology, telepresence services have emerged as an essential part of modern communication systems. Concurrently, the rapid growth of fog computing presents new opportunities and challenges for integrating telepresence capabilities into distributed networks. Fog computing is a component of the cloud computing model that is used to meet the diverse computing needs of applications in the emergence and development of fifth- and sixth-generation (5G and 6G) networks. The incorporation of fog computing into this model provides benefits that go beyond the traditional model. This survey investigates the convergence of telepresence services with fog computing, evaluating the latest advancements in research developments and practical use cases. This study examines the changes brought about by the 6G network as well as the promising future directions of 6G. This study presents the concepts of fog computing and its basic structure. We analyze Cisco’s model and propose an alternative model to improve its weaknesses. Additionally, this study synthesizes, analyzes, and evaluates a body of articles on remote presence services from major bibliographic databases. Summing up, this work thoroughly reviews current research on telepresence services and fog computing for the future.

## 1. Introduction

The sixth-generation (6G) network is anticipated to be the next generation of the 5G network, utilizing higher frequencies to transmit more data at faster speeds [1]. It is expected to outperform the fifth-generation (5G) network in various technological areas, making it crucial for bandwidth-intensive applications such as 8K video streaming, virtual reality (VR), and augmented reality (AR) [2,3]. The new network will have lower latency, making it more suitable for applications that require real-time data synchronization, such as autonomous driving and cloud computing (CC) [4]. The 6G network will also incorporate advanced technologies like artificial intelligence (AI) and machine learning (ML) to enhance network efficiency and reliability [5]. The 6G network is expected to be a groundbreaking advancement in mobile technology, potentially transforming our interactions with the world.

The development of the 6G network is already underway, with widespread implementation expected by 2030. Early research in this area includes the International Mobile 2030 project [1,6]. Researchers are working diligently to ensure that the 6G network surpasses the capabilities of previous generations, meeting future quality of service (QoS) requirements [7]. Like previous generations, 6G will have unique application characteristics and technology trends that will define new performance requirements and significantly change the standard of service of the 5G network. As with 5G network deployments, the 6G network will face many new challenges [8]. One major challenge researchers have pointed out since the development of the 5G network is the development of commercial transceivers operating at THz frequencies [9]. In this regard, the formation of the future 6G network, its main services, and its core technologies are topics scholars have studied actively. Table 1 compares the potential key performance indicators of 6G and those of 5G [10,11].

### Methodology and Work Structure

This paper presents research on the development of the 6G network, analyzing its future directions and range of services. The research is based on a comprehensive review of major bibliographic databases, such as Science Direct, IEEE Xplore, and Google Scholar as well as the selection and analysis of information from authoritative scientific publications. The paper used a dataset of recently published articles to cover the rapid development of 6G technology. The study focuses on three main areas: the future development and shaping of the 6G network, the services it supports (including telepresence and fog computing), and their recent development.

Additionally, the study presents an overview of the services that 6G technology aims to offer users. Specifically, we concentrate on the telepresence service, which is of significant interest to researchers and has the potential for widespread application in various fields. We collected over 700 articles related to the keyword “telepresence” from the IEEE Xplore database in a short period from 2020 to the end of 2023. The articles were analyzed to identify the main research directions in the field of telepresence. Telepresence can potentially revolutionize various aspects of our lives, including education, health, and entertainment. We use the following multistep approach to ensure a thorough investigation of existing research.

1. *Database selection*: The literature was gathered from major digital libraries, including IEEE Xplore, ACM Digital Library, Springer, Elsevier (ScienceDirect), and Google Scholar. These sources were chosen because they cover computer networks, fog computing, and telepresence technologies extensively.
2. *Search strategy*: A set of predefined keywords was used to retrieve relevant studies. The primary keywords included “telepresence services”, “fog computing”, “edge computing”, “remote presence”, “5G and 6G networks”, and “distributed computing models”. Boolean operators (AND, OR) were used to refine the search results.
3. *Inclusion and exclusion criteria*: we considered the following inclusion and exclusion criteria:
  - Inclusion criteria: peer-reviewed journal and conference papers published within the last 4 years, articles focusing on fog computing applications in communication networks, and studies discussing telepresence architecture.
  - Exclusion criteria: papers lacking technical depth, works unrelated to telepresence and fog computing, and studies focused solely on cloud computing without fog or edge-based considerations.
4. *Screening and selection*: After retrieving the initial results, duplicate papers were removed and abstracts were reviewed for relevance. Selected studies were then analyzed based on their contributions to telepresence and fog computing integration.
  We considered the following approaches:
  - *Thematic analysis:* the literature was categorized into key themes, including fog computing architectures, Cisco’s fog model, security challenges in telepresence-fog integration, and potential improvements for next-generation networks.
  - *Comparative evaluation:* Cisco’s fog computing model was assessed based on identified weaknesses and an alternative model was proposed to address these limitations.
  - *Emerging trends and future directions:* the survey examines technological trends that influence the development of telepresence services in 6G networks, highlighting promising research directions.

The research is presented in the following structure: Section 2 discusses the development of 6G technology and the services it can provide in the future. Section 3 focuses on fog computing technology. Section 4 provides an overview of telepresence services’ development and basic concepts. Section 5 discusses the challenges of using fog computing technology to support telepresence as well as promising research directions. Section 6 presents the paper’s conclusions.

## 2. 6G Network Development and Key Service Areas for the Future

**Figure 1:**
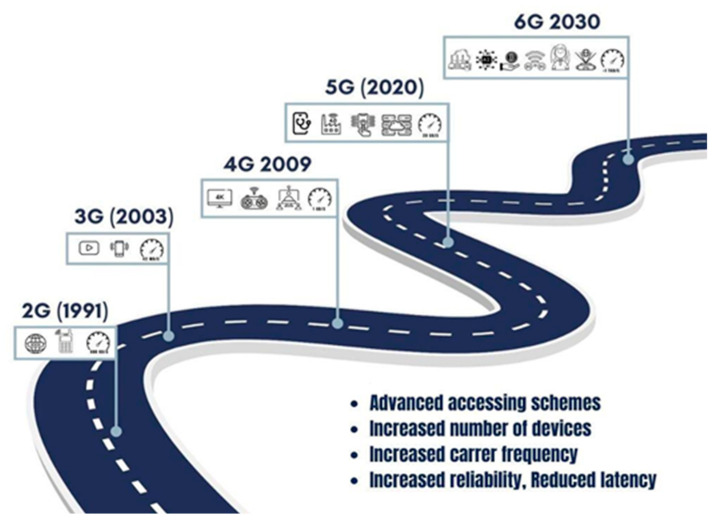
A brief overview of the history of wireless communication networks.

Fifth-generation networks are steadily progressing toward ultralow latency applications like drone networks and haptic Internet [12]. However, the exponential growth of data-driven automation might outrun even the capabilities of advanced 5G systems. This underscores the need for next-generation networks, e.g., 6G. We anticipate the arrival of 6G communication networks around 2030 [1]. Figure 1 presents a concise overview of the history of wireless communications networks, including 6G [1,13].

The primary research challenge is achieving data transmission rates of up to 1 Tbps per user. Utilizing the sub-THz and THz spectrum bands efficiently is crucial to achieving this goal [10]. Another significant challenge involves integrating and implementing smart technologies, diverse data sources, AI, and telepresence services. This presents a multitude of technical and mathematical hurdles on the path to 6G. Given these complexities, the 6G network remains a major area of ongoing research.

### 2.1. Fundamental Changes in the Development of 6G Communication Networks

The development of communication networks is one of the key factors in the development of modern society. Communication networks connect people, devices, and systems, enabling us to communicate, work, and access information. Each new generation of communication networks brings with it new opportunities and benefits. Fifth-generation networks are currently being rolled out and delivering significantly faster data speeds than previous generations. However, technology development is not stopping; research is already underway to develop 6G networks [14,15]. Fundamental changes in the development of 6G networks will involve several key areas:

- Ultralow latency communication networks. New technologies have led to even more significant changes in communication network construction, as it required data transmission with a delay of 1 ms, which is currently 100 times less than in existing communication networks. At the same time, it is worth noting that the concept of a 6G network leads to such a process as the decentralization of communication networks, as the fundamental limitations on the speed of light transmission are insurmountable [12,16].
- Ultradense networks. Ultradense networks are one of the key characteristics of 6G networks and will only become more important as the technology evolves. It is already predicted that by 2030, there will be around 1 million Internet of Things and other devices per square meter of floor space [17]. This means that communication networks must be able to support a huge number of low-latency, high-bandwidth connections. There are several ways to achieve ultradense networks. One is to use new frequency bands. A millimeter-wave communication network using 30 to 300 GHz frequencies is a better alternative. As the frequency increases, the millimeter-wave channel becomes more susceptible to shadowing and tropopause effects of millimeter-wave propagation [18]. However, due to the extremely short wavelength, high beam gain can be achieved by using a large number of antennas. This will solve the problem of loss and shadowing by tuning the signal power in the desired direction using multidirectional antennas [19]. Another way to achieve ultradense networks is by using new technologies such as AI and ML. These technologies can help optimize the use of spectrum and network resources, allowing more devices to be connected without compromising performance [20].
- Internet of skills. The Internet of Skills is a concept that emerged in 2017. It involves the use of ultralow latency networks to provide new services that will allow people and robotic devices to learn new skills. The basic idea behind the Internet of Skills is that ultralow latency networks will enable the real-time transfer of skill model data. This will allow new skills to be taught to humans and robots without the need to be physically present in one place. For example, the Internet of Skills could be used to teach people new languages, equipment skills, or creativity. The Internet of Skills can also be used to teach robots new tasks, such as car repair or surgery [21,22].
- Flying networks. Flying networks are another fundamental change in the development of communication networks. They combine flying and terrestrial segments of networks into a single communication network. Flying networks have several advantages over traditional networks. They allow for coverage even in hard-to-reach areas and higher data transmission speeds. One of the key features of flying networks is ultralow latency. This is because the distance between the flying device and the subscriber can be relatively short [23]. Flying networks have great potential for the development of new applications and services. They can be used to provide emergency communications, to monitor and manage infrastructure, and to create new entertainment. Flying networks have become one of the main segments of the 6G integrated networks of the 6G concept or space–terrestrial integrated network (STIN), which was approved by ITU-T [24,25].
- Unmanned transport. One of the key areas of development of unmanned transport is networking with roadside computing facilities. This will allow unmanned transport to access information about road and environmental conditions over long distances. This will contribute to the safety and efficiency of unmanned transport. Here are some specific examples of how networking unmanned vehicles can be utilized.
  - Warning of potential hazards: Unmanned vehicles can receive information about roadworks, accidents, and other hazards that are out of sight. This will allow them to take action to prevent collisions or other incidents [26].
  - Traffic optimization: unmanned vehicles can coordinate their movements with each other to avoid congestion and increase the efficiency of road use [27].
  - Improved customer service: unmanned vehicles can provide personalized services to customers, such as offering routes tailored to their preferences or needs [28].

With these changes, the 6G network will significantly alter our lives. Everything promises big changes in the near future, from industries to remote services, from healthcare to education.

### 2.2. Main Trends in the Development of 6G Networks

Based on the analysis of fundamental changes in the development of communication networks, it can be argued that by 2030, they will become ultradense, with ultralow latency. This will reduce the digital divide between territories, as the network will be decentralized. The widespread adoption of the Internet of Things and smart cities will contribute to the ubiquitous service capability of 2030 networks [29]. In addition, 2030 networks will acquire several new characteristics through advances in network and communication system technologies and related industries. For example, they will become more energy-efficient, reliable, and secure.

#### 2.2.1. Telepresence Personalization of the Network

In an era of rapid technological development, telepresence is becoming an inevitable trend, helping to overcome geographical boundaries between people. Telepresence refers to the use of technology to create the perception or sensation of being physically present in a remote location. This is achieved through telerobotics, high-fidelity video communication, and immersive interfaces that enable real-time interaction, even when participants are geographically distant [30,31]. Telepresence applications use an avatar to simulate and perform operator actions at a distance. Telepresence makes it easier for people to connect wherever they are in the world. In an era of rapid technological development, telepresence is becoming an inevitable trend, helping to overcome geographical boundaries between people.

Telepresence applications use an avatar to simulate and perform operator actions at a distance [30,32]. First, a new type of communication, holographic type communications (HTC), is defined for telepresence in 6G networks. This type of communication allows for increasing the level of immersiveness of human interaction through the network and within the framework of human–robot communication [33].

The future networks will be populated not only by data but also by virtual entities that can think and act independently. These entities, or avatars, will allow people to interact with the world from anywhere [34]. For avatars to fully interact with the world around them, very low-latency networks must be provided. Only then will the person controlling the avatar be able to feel like they are on the ground (deep level of immersiveness) [35].

In the future, as the population of avatars increases and their functionality improves, there will be situations where a single user can manage multiple avatars simultaneously. This can be carried out through the main avatar or otherwise. Such avatar networks may become one of the most important traffic sources in 2030 networks [36]. They will support new types of interactions between humans and avatars that require ultralow latency networks.

#### 2.2.2. Flying Networks

Some works have proposed the convergence of terrestrial and satellite networks to fulfill the requirements of 2030 communication networks [37]. However, the advantages of satellite networks in terms of coverage are offset by disadvantages in terms of delay. The latency in satellite networks is around 250 milliseconds, which may not be acceptable for some applications [38]. The orbital altitude of low-orbit satellites is 700–1500 km. The convergence of terrestrial and satellite networks cannot provide the ultralow latency required for some applications such as telepresence [39]. Latency in satellite networks is around 250 milliseconds, which is unacceptable for such applications [40]. More realistic scenarios for building 2030 networks are the use of unmanned aerial vehicle (UAV)-based aircraft or even the use of tethered UAVs that can operate at altitudes of tens and hundreds of meters [41]. This is especially important for areas with low population density and hard-to-reach areas. At the same time, the low-orbital satellite constellation segment can be used to host remote orchestrators and virtual function repositories, where, in this way, the issues of availability of such services for hard-to-reach and remote areas can be resolved. At the same time, the active switching and computing part will be formed by airborne (UAVs and tethered balloons) segments [42].

The structure of the ultralow latency communications network in 2030 includes elements to support a wide range of applications requiring latency of one millisecond or less [12]. These applications include telepresence (including HTC), avatars, drones, medical networks, AR, industrial humanoids, and applications to support cloud-managed Internet of Things (IoT) concepts.

#### 2.2.3. Nanonetworks

By 2030, the various applications of nanogrids and nanovehicles are expected to find widespread use [43]. Advances in spectroscopy have made infrared microspectrometers affordable, and they can be embedded in smartphones. This means that every user can obtain daily analyses of products and other items of interest for rapid quality confirmation. Such analyses are performed by obtaining a spectrogram and quickly analyzing it in the appropriate cloud [44]. It can be argued that such services will be widely used and will create significant new traffic on communication networks.

Infrared spectrometers make it possible to use pheromones to receive information, as theorized earlier. Pheromones are already used to transmit information, but their reception so far could only be carried out by living organisms [45]. In particular, the development of nanonetworks and nanovehicles will further increase the density of networks. This will be because nanovehicles can be placed in hard-to-reach places, such as inside buildings or the human body [46]. Advances in spectroscopy will make it possible to use infrared microspectrometers in smartphones. This will allow users to analyze products and other items anytime and anywhere. Such services will be widely used and create significant new traffic on communication networks. The use of infrared spectrometers will also allow pheromones to receive information [47]. This opens new possibilities for interaction between people and devices. For example, pheromones could be used to control drones or to create virtual assistants.

Thus, by 2030, communication networks will have a fundamentally new architecture and be capable of providing a wide range of services based, among other things, on the use of nanonetworks and nanomaterials [48].

## 3. Fog Computing—A Promising Technological Solution for the 2030 Networks

### 3.1. A General Overview of Fog Computing

Before the advent of cloud computing, enterprises had to invest in their own storage and processing hardware and software. This led to many difficulties in scaling up, power consumption, maintenance, and support costs for these storage and processing systems. Cloud computing has emerged as an inevitable development that has solved some of these problems. With cloud computing, businesses can rent storage and processing services from external vendors. Cloud computing has two main advantages. Firstly, they save money by sharing resources. Secondly, they help accelerate innovation by reducing the cost of deploying and operating applications [49]. It helps to save much more compared to the old method of storing and processing data, improves scalability, and reduces energy consumption.

However, cloud computing has limitations, such as latency and security. In the case of cloud computing, the request has to be sent to a remote data center, which can lead to significant delays. Fog computing is an emerging technology and has the potential to address some of the shortcomings of cloud computing [50]. Data storage and processing services are placed closer to the end users in fog computing. Storage and computing devices are increasingly appearing at the edge of the network. They are called fog nodes and can be owned and operated by end users or service providers [51].

An embedded processor on an IoT smart device board, a server in an ISP’s regional office, or even processing servers located in local offices or customer homes can all act as fog nodes, according to the OpenFog project [52]. This development is being driven by the expansion of the IoT, which is expected to cause an enormous increase in the number of linked devices. These devices are expected to generate around 79.4 zettabytes of data, with current estimates putting the number between 25 and 50 billion [53,54].

Fog nodes have a big advantage over data centers: they are closer to end users. This means that cloud services can be migrated from the network’s core, resulting in lower latency. Fog nodes can also be connected to the network via wired or wireless links. This allows them to be deployed in various locations, including remote and hard-to-reach areas. However, distributing cloud services to fog nodes is a complex task. Many factors need to be considered, including latency, energy efficiency, scalability, and cost [55].

Obviously, with the rapidly increasing development speed of connected devices, the commercialization of the 5G network and the exploration of the long-term vision of the 6G network are inevitable. With the 6G network, a new generation of applications and services (e.g., VR, telepresence, etc.) will be deployed and a large number of devices will be enabled [56]. Due to the expectations of 6G network advancements such as a 0.1 ms latency threshold and 10× improvement in energy efficiency, research on future fog computing architectures is urgent because if all data from the end device are processed by clouds directly at the edge, speed and efficiency will increase significantly [57].

### 3.2. Defining Fog Computing

Fog computing is a paradigm for network design that includes cloud platforms and client data centers as nodes along the path from data production to storage. Fog computing is an intermediate layer in the distributed networking environment that is highly interdependent with cloud computing and the IoT. Fog computing is based on the idea of a decentralized network that connects these two domains. Another way to understand fog computing is through a model that provides computing, networking, and storage resources through distributed servers at the edge of the network near the end users [58]. In a fog network, servers located at the edge of the network, near end users, are called fog nodes. Using fog devices, clients can obtain real-time responses for latency-sensitive applications.

Fog computing was first introduced by Cisco [59], and other researchers and industry representatives have defined fog computing from different perspectives. Another definition of fog computing was proposed by Yi et al. [60]. It is defined as “a geo-distributed computing platform with a set of requirements including a variety of computing devices connected to the WAN at the edge of the network and not fully supported by cloud computing services to jointly provide elastic transmission, storage, and computation in remote environments to a large number of users in close proximity” [60].

### 3.3. Characteristics of Fog Computing

The essence of fog computing is to extend cloud computing but move it closer to the end devices. Fog computing moves storage, networking, and computing services closer to users, acting as an intermediary between users and data centers. Servers at the network’s edge, close to users, are called fog nodes [61]. They can be placed anywhere with a network connection. Any server capable of computation, network connectivity, and storage can be called a fog node. Developing a fog computing framework enhances the data processing capabilities of organizations. With fog computing, data can be processed wherever it is most efficient, leading to reduced latency and improved performance. It is important to process data quickly in some applications, such as manufacturing. For example, connected machines must be able to react to failures as early as possible [62].

Fog computing allows for low-latency networks between devices and analysis points. This is achieved by placing computing resources closer to where the data is created. This design reduces the need to transmit data over long distances, resulting in lower latency and improved performance. Additionally, fog computing can be used when high-speed networks are unavailable [61]. In such cases, data can be processed locally, which can be especially important for applications that require fast response times. Yi et al. [60] argue that fog computing offers many new and useful features compared to cloud computing. These features can be categorized as follows [61,62,63,64]:

- Flexibility: Fog computing provides distributed storage and computation resources that can work with various end devices, such as networked sensors, industrial machines, and wearable devices. This makes the fog cloud suitable for various IoT applications, from environmental monitoring to manufacturing management.
- Real-time communication: Fog computing enables simultaneous communication between cloud nodes. This is essential for IoT applications that require a proactive response, such as security monitoring and traffic management.
- Physical distribution: Fog computing distributes applications and services, unlike cloud computing. This means they can be stored anywhere. This reduces latency and improves performance for IoT applications.
- Low latency and location: Fog computing is located near end devices, providing shorter latency when processing information. Additionally, it supports location responsiveness by storing fog computing nodes in multiple locations.
- Compatibility: Fog computing modules can interact with various platforms through different service providers, ensuring compatibility.
- Providing web analysis and cloud integration: Fog computing sits between the end devices and the cloud to play an important role in the ability to process and compute information near the end devices.
- Heterogeneity: Fog computing endpoints or nodes are designed by various companies, each with their provenance schemes, and must be stored according to their location requirements. This allows fog computing to be adaptable to different platforms.
- Ensuring flexibility: Fog computing requirements include connecting directly to devices, such as mobile devices. This allows the use of flexible technologies such as the Location and Identifier Sharing Protocol (LISP), which requires a distributed indexing system.

### 3.4. Structure of Fog Computing

Fog computing is a promising paradigm that shifts some computational operations from centralized data centers to the edge of the network, revolutionizing traditional data processing. Through the closer placement of computing resources to the data source, this method lowers latency, increases bandwidth efficiency, and improves overall system responsiveness. Fog computing makes it possible to integrate IoT devices seamlessly, analyze data in real time, and make faster decisions by utilizing edge devices. In addition to relieving the strain on central data centers, this distributed computing paradigm opens up fresh possibilities for cutting-edge applications across various industries, such as healthcare, industrial automation, smart cities, and driverless cars. It shares less storage, processing, and network resources between the CC data center and the end device [61]. Fog computing provides distributed architectures involving multiple devices and small data centers near data creation sources, such as sensors, mobile phones, and handheld devices. The architecture of fog computing is commonly structured around two primary models: the hierarchical and multitier models [61,65].

#### 3.4.1. Hierarchical Model of the Architecture

**Figure 2:**
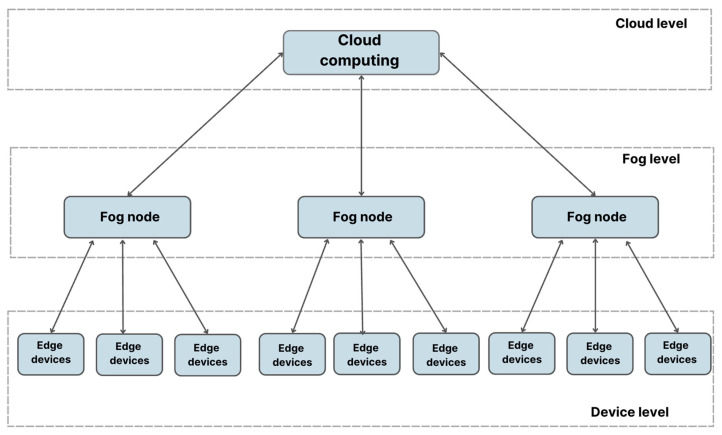
The main architecture of the hierarchical fog computing model.

In this design, fog computing ensures that services are delivered smoothly over the device-fog-cloud pathway by mediating between the end and remote cloud computing layers [66]. Figure 2 shows the functioning and structure of the fog computing architecture’s hierarchical model.

(a) Terminal layer (Terminal)

All over the system’s coverage area, end devices of all shapes and sizes make up the end-device layer. Each of these devices, from sensors and smart cards to controllers and smart automobiles, is essential to the network’s data collection, transmission, and processing operations. As the principal data generators and interfaces with the physical world, they are integral to the system’s architecture. Their decentralized nature allows for all-encompassing coverage as well as the ability to monitor and control several applications and domains in real time. These devices are commonly organized into groups based on their specific functionalities and roles within the system. At the end-device layer, their main tasks involve the collection and transmission of data to the upper layer for subsequent processing and analysis. Furthermore, with the advancements in computer technology, end devices are now capable of performing localized tasks. In numerous Internet of Things systems, the upper layer comprises controllers that receive commands and control from the cloud to make decisions [67].

(b) Fog layer

The local cloud layer consists of a variety of physical devices, such as routers, gateways, access points, base stations, and local cloud servers. These components form the backbone of the local cloud infrastructure, facilitating efficient data processing and storage closer to the edge of the network [62]. Local cloud nodes are deployed in various locations within the system coverage area, depending on the application. For example, they may be located on a production floor, a power grid pole, next to railway tracks, inside a car, or on an oil platform. Local cloud nodes can be either static, such as those found at bus stops or coffee shops, or mobile, such as those installed inside moving vehicles. These nodes provide services to end devices and can also compute, transmit, and temporarily store data. To enhance their computation and storage capabilities and enable communication and cooperation with the cloud, IP network cores have been developed [68].

(c) Cloud layer

The cloud layer consists of high-performance computing devices and data servers with large storage capacity. Its purpose is to perform computation, analysis, and long-term data storage for users. At this level, users have access to a wealth of cloud computing resources housed in massive data centers with enormous storage capacities and powerful processing capabilities. The backbone of cloud computing is data centers, which provide on-demand, scalable computer resources. The cloud performs two functions inside a fog computing architecture: storing backup copies of data and providing persistent storage. To make data accessible and prevent data loss, users often cache and store it in the cloud when it is not immediately needed. By bringing together fog and cloud computing, we can improve resource usage, make data more resilient, and scale easily to meet changing demands.

#### 3.4.2. Proposed Multitier Fog Computing Model

Traditional cloud-based architecture often fails to meet the demanding needs of real-time, high-fidelity telepresence, especially when it comes to holographic and telerobotic interactions. Fog computing addresses the following main issues with telepresence applications.

- Processing video streams, spatial audio, and sensor data closer to users to minimize round-trip delays.
- Preventing network congestion through local data filtering, compression, and adaptive streaming.
- Improving reliability, which is vital for uninterrupted telepresence experiences.
- AI-driven predictive caching and adaptive resource management boost responsiveness in ever-changing network conditions.

Upon analysis of Cisco’s proposed fog computing model, several issues arise. Firstly, the model lacks a clear definition of fog aggregation nodes, leaving ambiguity as to their location—whether at mobile network base stations, data centers, or elsewhere. Secondly, the computing resources are undefined, making it difficult to assess the model’s efficiency and scalability. The model does not specify who will be responsible for deploying and managing the fog aggregation nodes. Additionally, Cisco’s fog aggregation model has several other limitations. Data analysis on fog nodes may result in higher latency compared to data analysis in data centers. Storing data on fog nodes may increase the risk of cyberattacks and managing a large network of fog nodes can be complex. Due to these limitations, the Cisco fog model has not been widely adopted. Nevertheless, this model has the potential to address latency and bandwidth issues in IoT applications [69].

**Figure 3:**
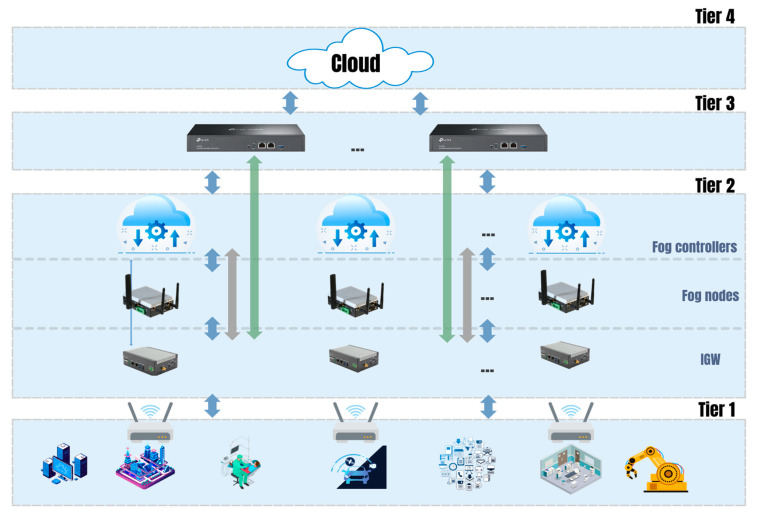
Four-tier fog computing architecture.

Several studies have proposed layered fog models to address the limitations of the Cisco fog model [70]. These models divide fog nodes into different layers based on functionality and capabilities. This work proposes a four-layer fog model for deploying IoT applications and other applications on the 6G network. This model reduces latency for IoT applications and improves resource efficiency, scalability, and security. The model is easy to deploy and manage, as illustrated in Figure 3. Devices in the two neighboring layers communicate with each other using wired or wireless connections. Each device supports multiple virtual machines within the available resources. The cloud, fog controller, and fog node each have multiple virtual machines to meet the computational requirements of various applications.

***Layer 1: SDN controller.*** The SDN controller plays a crucial role in managing and coordinating data flows in the four-tier fog computing model. It performs key functions, including managing data flows between the fog and cloud layers, ensuring efficiency, and optimizing the use of network resources. Additionally, it monitors and controls the entire fog network, including IoT devices, fog nodes, and fog controllers [71]. The SDN controller also plans and optimizes the fog network to meet the needs of Internet of Things applications. By utilizing advanced technologies like SDN, NFV, and AI, SDN controllers offer practical benefits such as improving network resource utilization efficiency, reducing latency for IoT applications, enhancing the ability to extend and secure your network, and simplifying network deployment and management [72].

***Layer 2: IoT networking.*** The IoT gateway receives data from devices and performs initial filtering and processing before forwarding them to the appropriate fog node or fog controller. The fog node processes low-latency IoT applications, stores data locally, and provides computing and storage services to IoT devices. This layer handles low-latency processing, data caching, and protocol translation (e.g., converting raw holographic data into compressed formats). Furthermore, the fog controller oversees the fog nodes, assigns resources to IoT applications as required, and coordinates tasks to enhance the performance of the entire system. In case of a failure, for instance, the fog controller can redirect data to another fog node to ensure uninterrupted service.

***Layer 3: IoT and coordination devices***. Layer 3 comprises IoT devices and coordination devices, including wearable devices, smart cameras, VR/AR headsets, and robotic controllers, that collect data and control devices in the fog network. IoT devices gather data from the environment and transmit it to the IoT gateway for further processing. In telepresence applications, such devices capture high-resolution video, spatial audio, and biometric data for remote interaction. Coordination devices establish virtual clusters based on the location of IoT devices, which optimizes management and service delivery for devices in the same area.

***Layer 4: Cloud and the benefits of a four-tier model.*** The fourth tier is the highest tier in the four-tier fog computing model. Its main functions include long-term data storage, data analytics services, and support for IoT applications with high latency.

The considered fog computing model should employ advanced compression techniques, transport protocols, and AI-driven optimizations to manage high-bandwidth and real-time data transmission. This includes the following.

(a) Compression algorithms for efficient data transmission
  - H.265/HEVC, AV1, and versatile video coding (VVC) can be deployed to reduce the bandwidth required for high-definition and holographic video streams [73,74].
  - Point cloud compression (MPEG-I HoloCodec, Draco) can be used to optimize 3D holographic rendering for telepresence [75].
  - Edge-based adaptive streaming can ensure that content quality adjusts dynamically to network conditions [76].
(b) Latency-aware transport protocols
  - Quick UDP Internet connections (QUIC) can be used to minimize connection latency for real-time communication [77].
  - WebRTC enables peer-to-peer holographic streaming for decentralized telepresence [78].
  - 6G-integrated network slicing can dynamically assign dedicated bandwidth to telepresence applications [79].
(c) AI-driven resource optimization [80,81]
  - Reinforcement learning-based scheduling can allocate computational resources dynamically based on network congestion and user demand.
  - Edge AI inference acceleration can be deployed to reduce dependency on cloud-based processing, improving response times.
  - Predictive caching and prefetching can optimize holographic data delivery for a seamless experience.

The proposed fog computing framework leverages different mechanisms to assist QoE of telepresence services. NFV and SDN are deployed to enable flexible, on-demand bandwidth allocation over the proposed fog structure for latency-sensitive telepresence tasks [82]. This ensures mechanisms for supporting dynamic load balancing across multiple fog nodes. AI/ML can also be introduced to monitor QoE. ML models can predict network congestion and dynamically adjust resources, and anomaly detection algorithms can proactively mitigate network failures. As a clear contrast, Table 2 presents a comparison between the Cisco fog model, edge–cloud hybrid architectures, and the multitier fog model proposed in this report. The comparison is insightful in nature with respect to parameters such as latency, energy efficiency, scalability, and security.

**Figure 4:**
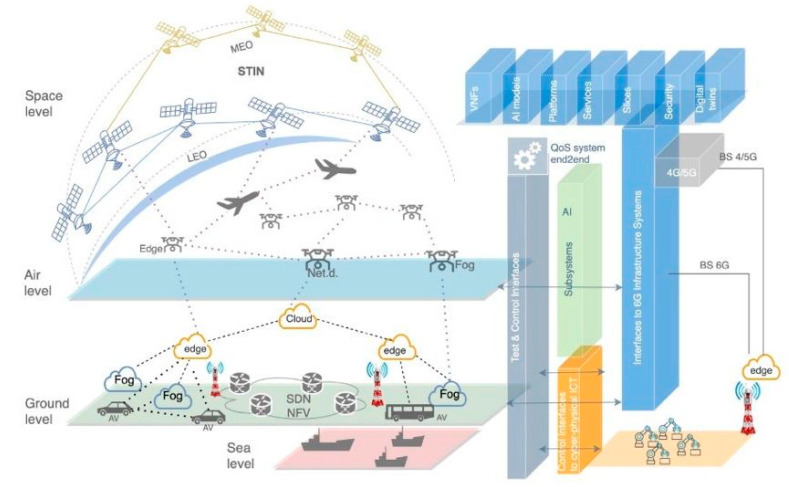
Integrated architecture of multidimensional earth–sea–air–space.

Researchers are continuously developing new network architectures to clarify the significant changes brought by the 6G integrated land–sea–air–space network, which transcends traditional boundaries and brings people into a new era of communication and interaction [83,84]. This paper proposes an integrated architecture based on distributed edge and fog computing, utilizing AI and distributed edge computing to achieve seamless communication, continuous coverage, and efficient resource allocation [85]. The architecture is illustrated in Figure 4.

Each new network architecture has unique advantages that contribute to the overall development of communication technologies. Fog computing is a paradigm gaining recognition and adoption due to its contribution to modern computing technologies. It promises potential benefits and applications. However, successful implementation requires addressing challenges such as security, standardization, and governance. With appropriate investment and development, fog computing could play a crucial role in creating a smarter, more secure, and more efficient network in the future.

## 4. Fog Computing Application for Telepresence Services

Telepresence technology is an area of research with a wide range of applications in industry, education, and commerce. The question of how this technology will evolve in the future is also discussed. This work reviews the developments, achievements, and challenges facing this field and analyzes the general trends in telepresence technology. This article explores the application of cloud computing at the edge of the services provided by next-generation networks. It covers improvements in image and sound quality, the introduction of visual interfaces, and the use of robots to replace humans in remote operations. Specifically, it examines how cloud computing at the edge can be applied to telepresence services.

### 4.1. Analysis of the Recent Development of Telepresence Services

Telepresence technology has the potential to revolutionize communication and interaction in the future. It enables real-time virtual meetings with colleagues or friends from anywhere in the world as if they were in the same place. This is made possible through a combination of hardware and software that allows for high-quality audio and video transmission over a network. Telepresence technology has become widely adopted due to advances in technology and modern communications. It is an alternative to traveling long distances for conferences or meetings, saving time and money and reducing environmental damage. Additionally, telepresence technology enables remote monitoring and management of various tasks.

Furthermore, telepresence has the potential to enhance quality of life by offering educational and healthcare support services in remote and rural areas where access to technological advancements is limited. Additionally, in the context of automated systems, telepresence can incorporate human elements, such as operators and users of various applications, to provide or receive similar services without physical constraints that may compromise the quality of the services provided. Recent advances in science and technology have led to significant improvements in telepresence [86]. This technology has revolutionized the way people communicate and interact over long distances. Real-time interaction is now possible, allowing for deeper immersion and connection despite physical distance. These improvements have been made possible through advancements in video quality, audio transmission, and the development of an intuitive user interface [87].

Advances in high-definition cameras and improved compression algorithms have enabled the transmission of higher-quality video, resulting in a more realistic and clearer experience. Additionally, the introduction of 3D screens, including technologies to realize HTC terminals, has taken the immersive experience to a new level, allowing people to feel as if they are physically present in a remote location [88]. For successful telepresence, it is essential to have clear audio with noise-cancellation capabilities. Recent advancements in this field have helped to eliminate echo and ambient noise, resulting in improved sound quality and more natural, seamless conversations. Additionally, an easy-to-use and understandable user interface is crucial for effective telepresence. Advances in interface design have resulted in the development of an intuitive user interface that simplifies the interaction and control of telepresence devices. Users can now access certain functions with simple gestures or voice commands, using devices that are smoother and more convenient.

The history of telepresence technology dates back to the 1960s, when simple camera and screen systems broadcast static images of users. Today, with the advancements in technologies such as VR, AR, AI, and ML, the potential of telepresence has become even more evident [89]. This technology is increasingly prevalent and is expected to become essential to our daily routines.

This study exemplifies the main stages in the development of telepresence. Initially, telepresence involved a video-based virtual space encounter that used multiple cameras to gather quantitative and in-depth information [90]. Since the 2020s, telepresence has become a popular technology used in various fields, including education, healthcare, and business.

### 4.2. Application of Fog Computing to Telepresence Services

Nowadays, the development of remote services, including telepresence, faces various threats and challenges, such as network latency, instability, and security against network attacks. Latency is a crucial factor to consider when designing and developing any remote-control system. Latency and instability can negatively impact the transmission of control signals and sensory information between the operator and remote device in a remote-control platform. This leads to decreased stability and efficiency in completing tasks.

Typical telepresence techniques involve using cameras to track the operator’s head movements, stereoscopic vision, audio feedback, force feedback, and touch sensors. For ideal telepresence, it is necessary to transmit all human senses from a remote-controlled location to the operator’s location. A three-dimensional image combined with stereo sound is required to record physical presence. To enable a three-dimensional holographic display, an extremely reliable data network capable of handling about 4.32 Tbps of data is required [91]. Additionally, the latency requirement must be less than a millisecond to ensure synchronization of multiple viewpoints. These communication constraints will be significant.

Cloud computing is already used to support data transmission, storage, and processing. It has partially solved the problem of centralized data center storage and computing through the network core. However, researchers have found that the rapid growth of mobile devices is a challenge faced by telepresence technology in recent years. Researchers predict that global mobile traffic will increase by 670 times more than in 2010 by 2030, primarily due to the development of machine-to-machine (M2M). This article provides an overview of the challenges, technologies, and applications from 5G to 6G. This increase in traffic makes it challenging to process data directly using cloud computing. As a solution to this issue, cloud edge computing (fog computing) has emerged as a new technological solution. Cloud services are now being pushed from the core to the edges of the network, significantly reducing latency and improving remote presence applications’ performance. This is particularly important for real-time applications, such as video conferencing and online gaming. Additionally, cloud computing at the edge can easily scale to meet the needs of growing remote presence applications. The application of cloud edge computing to 5G and 6G mobile network services is a promising development direction that has attracted the attention of researchers.

**Figure 5:**
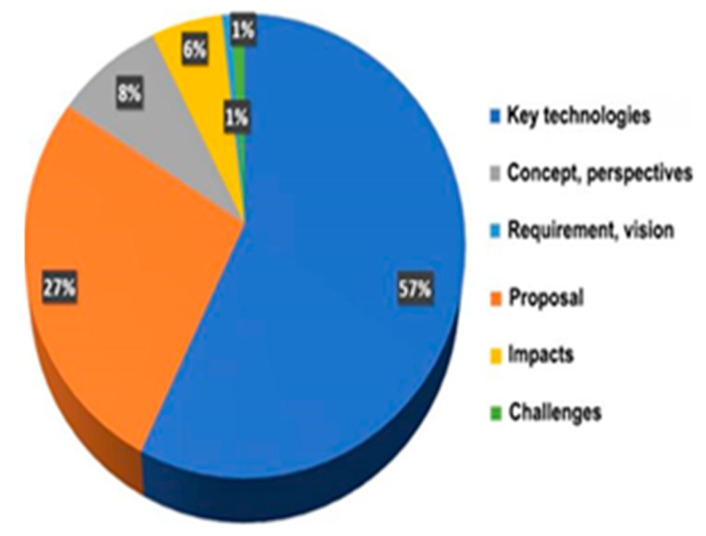
Pie chart showing the percentage distribution of research studies by topic.

As part of this article, we conducted a small survey. We used search data from the IEEE Xplore Science and Technology website to objectively evaluate research related to telepresence technology and present the most accurate viewpoint based on the data collected. To our surprise, we found over 700 studies, including conferences, articles, and journals, when searching for the keyword “telepresence” during the rapid growth of telepresence technology from 2020 to the end of 2023. The most common research areas were key technologies, applications, concepts and development directions, telepresence technology impact, vision and requirements, and current challenges. Table 3 and Figure 5 provide a summary of these studies.

Upon analysis of the presented studies, it can be concluded that research in the field of telepresence is primarily focused on two areas: telepresence applications and basic technologies. The development of robots for medicine, education, and other fields where they can replace humans receives the most attention. Our work indicates that research on the application of fog computing in telepresence is currently at a very low level, almost nonexistent.

The rapid growth of global mobile traffic has led to the development and application new technologies, such as fog computing, to address network challenges like latency and instability. It is important to note that while significant academic attention has been given to research on fog computing, there has been just as much investment and focus on the application of fog computing in the IoT and haptic Internet. However, there has been limited research on the application of fog computing to the services provided by 5G and 6G mobile networks, such as telepresence services. Therefore, further research and investment in applying fog computing technology to telepresence services for 5G, 6G, and subsequent generations of networks may be necessary.

### 4.3. Security Issues with Fog Computing for Telepresence Services

Integrating fog computing for telepresence services raises many security challenges due to the inherently distributed nature of fog nodes, real-time data transmission, and the management of sensitive personal and enterprise data. Other security challenges include the following [92,93].

(a) As telepresence services rely on real-time data streaming, fog nodes become potential targets for man-in-the-middle (MITM) attacks.
(b) Decentralized fog nodes require robust access control to prevent unauthorized data access.
(c) IoT devices and endpoints involved in telepresence sessions may be compromised due to weak authentication mechanisms.
(d) Attackers can exploit vulnerabilities in fog computing infrastructure to overload nodes and disrupt telepresence communications.
(e) Malicious actors may alter real-time communication streams, leading to misinformation and privacy breaches.

Table 4 provides some key security concerns and the potential mitigation strategies to tackle these security challenges. Telepresence services require strong privacy-preserving techniques, especially when handling sensitive audio, video, and biometric data. This can be achieved through the following.

- Using federated learning to train user data without centralizing, it reduces data exposure risks.
- Homomorphic encryption can enable computations on encrypted data without decryption, ensuring privacy in fog-based AI processing.
- Differential privacy adds noise to data queries, preventing user identity leakage.
- Decentralized self-sovereign identities (SSI) enhance privacy and control over personal data.

## 5. Standardization and Interoperability in Fog Computing and Telepresence Services

The rapid expansion of fog computing and telepresence services in 6G networks necessitates robust standardization and interoperability frameworks to ensure seamless integration among heterogeneous devices, platforms, and service providers. Given the diverse nature of telepresence applications, ranging from real-time holographic communication to remote medical consultations, establishing compatibility across different hardware and software ecosystems is paramount. Several standardization bodies and industry consortia have been actively working on defining frameworks for interoperability, including IEEE 1934 (fog computing and networking architecture standard) [94], ETSI multiaccess edge computing (MEC) [95], and 3GPP initiatives for edge intelligence and 6G [96].

The IEEE 1934 standard, formally named “IEEE Standard for Adoption of OpenFog Reference Architecture for Fog Computing”, has been published to provide a detailed standard for fog computing methodology. This standard aims to distribute computing, storage, control, and networking services prescribed near the end user, all situated on a cloud-to-thing continuum between devices. Fog computing can be described as the architecture of fog networking or fogging where most computing, storage, and communications are implemented locally through edge devices or fog nodes, thereby virtually eliminating route data through the backbones of the Internet. The IEEE 1934 standard adopts the OpenFog reference architecture, which focuses on eight foundational pillars to ensure a robust and interoperable fog computing environment [94]. Table 5 provides a detailed discussion of these eight pillars.

The IEEE 1934 standard describes a multilayer architecture with different necessary functions to provide good load balancing and interoperability of fog services. Table 6 provides the main specifications of the functional layers of IEEE 1934. This fog standard can significantly contribute to harmonizing fog computing with telepresence systems under 6G. Table 7 provides the key contributions of the IEEE 1934 standard in evolving 6G telepresence applications. While IEEE 1934 offers a promising framework for fog computing and 6G telepresence, several practical implementation challenges and open research questions need to be addressed. These challenges include the following [94].

(a) Although IEEE 1934 promotes open standards, many telepresence and 6G service providers utilize proprietary architectures, creating integration difficulties.
(b) Decentralized fog environments are susceptible to security threats, necessitating continuous updates to authentication mechanisms.
(c) Optimizing energy consumption in fog nodes while maintaining high-performance computing remains a key challenge.
(d) Further research is needed to enhance AI-driven workload allocation across hierarchical fog layers. This includes developing novel intelligent resource allocation and management schemes.
(e) Integrating emerging technologies such as terahertz (THz) communication, intelligent reflecting surfaces (IRS), and quantum-secure networks with IEEE 1934 is an open research area.

3GPP does not have a dedicated fog computing model but incorporates fog computing principles within its 5G and 6G architecture, particularly through MEC and service-based architecture (SBA). While 3GPP does not explicitly define fog computing, it integrates fog-like distributed computing. Table 8 provides the key components of fog computing mapped to recent releases of 3GPP. The key achievements of different releases of 3GPP in MEC and fog evolution are summarized as follows [96].

- 3GPP Release 16: Introduced MEC offloading, uRLLC, and network slicing.
- 3GPP Release 17: Enhanced AI at the network edge, advanced user plane function (UPF) functionalities, and service discovery mechanisms.
- 3GPP Release 18+ (Toward 6G): This version includes AI-native networks, zero-touch automation, and federated learning at the edge, aligning closely with fog computing principles.

Summing up, Table 9 provides a comparison between fog models of IEEE, 3GPP, and ETSI.

## 6. The Mind Map for Future Work

**Figure 6:**
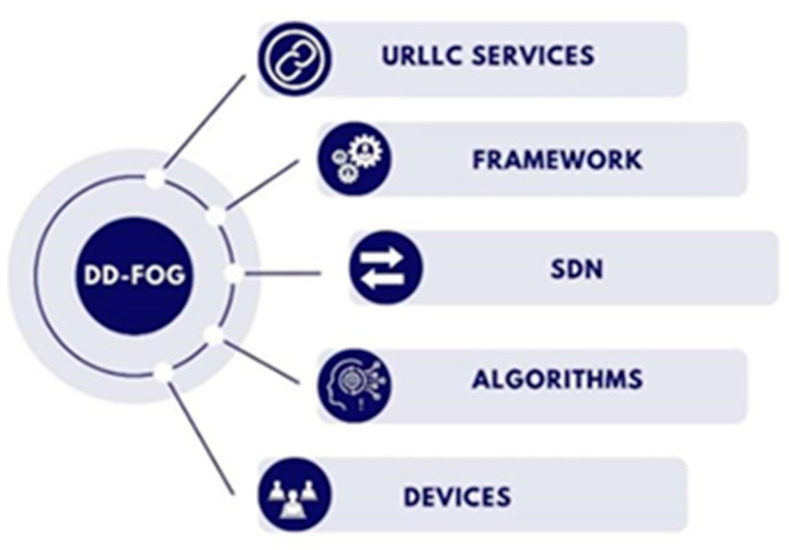
Research areas in fog computing.

The rapid development of technology has led to the emergence of many distributed endpoints and the creation of vast amounts of data that require due attention. In this context, the role of fog computing in supporting higher-end devices is undeniable. Therefore, there is an urgent need to study and research fog computing to develop and utilize all its advantages. Figure 6 shows some research areas currently of interest in various areas of fog computing. Moreover, Table 10 provides details on these future directions.

Future research in fog computing aims to create new frameworks that offer APIs and tools for the simple and efficient development, deployment, and management of fog computing applications. Additionally, the effectiveness of the developed frameworks will be evaluated based on criteria such as performance, scalability, security, and usability. The focus will be on the area of fog structures (Nebulas), which encompasses Fog2Fog migration, Nebula definition, and Fog2Fog networks. This research promises to contribute to the advancement of fog computing by providing new and efficient frameworks that will enable easy and efficient deployment and management of fog computing applications [99].

## 7. Conclusions

To summarize, this investigation has thoroughly examined the relationship between telepresence services and fog computing within the framework of advancing communication systems. An examination of recent academic advancements and real-world applications has provided insight into the advantages and difficulties of incorporating telepresence capabilities into distributed networks. The evolution of 5G and 6G networks, along with the rapid expansion of fog computing, has created opportunities for novel solutions to address the changing requirements of contemporary applications. This work contributed to the development of distributed computing infrastructures by examining the advantages and possible drawbacks of fog computing models, such as Cisco’s, and suggesting alternative ways. Furthermore, combining and assessing a wide range of the literature on remote presence services emphasizes the significance of ongoing research and advancement in this ever-changing subject. As we consider the future, the potential developments of 6G networks and the ongoing progress of fog computing have important consequences for improving telepresence technology. This work provided an overview of the main development directions for 6G networks. Summing up, this survey is a significant tool for scholars, practitioners, and policymakers who want to comprehend the changing environment of telepresence services and fog computing. This work contributes to the continued development of communication systems for the digital era by giving insights into existing trends, difficulties, and future directions, focusing on resilience and efficiency.

## Figures and Tables

**Table 1:** Potential KPIs of 6G compared to 5G [10,11].

KPIs	5G	6G
Peak data rate	10 GHz	1 THz
User experience data rate	1 GHz	>10 GHz
Bandwidth	1 GHz	100 GHz
Carrier bandwidth	400 MHz	Not defined
Channel	Random	Controlled
Operating bandwidth	Up to 400 MHz for sub-6 GHz bands Up to 3.25 GHz for mmWave bands	Up to 400 MHz for sub-6 GHz bands Up to 3.25 GHz for mmWave bands
Latency	1ms	0.1ms
Peak spectral efficiency	30 bps/Hz	60 bps/Hz
Artificial intelligence	Partial	Fully
Service level	Limited (VR/AR)	Widely (Tactile)
Max. frequency	90 GHz	10 THz
Mobility	500 km/h	>1000 km/h
Coverage	About 70%	>99%
Reliability	About 99.9%	>99.999%

**Table 2:** Comparison of fog computing architectures.

Feature	Cisco Fog Model	Edge-Cloud Hybrid	Proposed Multi-Tier Fog Model
Latency	Moderate (Higher than cloud but lacks defined aggregation nodes)	Low (Offloads computation to the cloud dynamically)	Very low (Optimized with hierarchical processing)
Energy efficiency	Moderate (Fog nodes process locally but lack power optimization strategies)	High (Cloud resources are used efficiently, reducing edge power consumption)	Very high (Efficient workload distribution across tiers)
Scalability	Low (Ambiguous fog node management)	High (Scalability depends on cloud resources)	Very high (Hierarchical fog architecture allows elastic scaling)
Security	Moderate (Decentralized data storage increases attack surface)	High (Cloud security mechanisms protect data)	Very high (Multitier security with edge encryption and access control)
Deployment complexity	High (Unclear management strategy)	Moderate (Requires cloud integration)	Low (Simplified due to multitier automation and orchestration)
Best use case	Basic IoT applications	AI-driven real-time processing	Ultra-low latency 6G applications (e.g., telepresence, industrial IoT)

**Table 3:** Classification of the considered studies by topic.

Topic of Research	Number of Studies
Key technologies	403
Proposal	190
Concept and perspectives	60
Impacts	40
Requirement and vision	6
Challenges	7

**Table 4:** Potential security concerns of fog computing for telepresence.

Security Concern	Mitigation Strategy
Data encryption	Implement end-to-end encryption (E2EE) using AES-256 or homomorphic encryption.
Access control and authentication	Utilize zero trust architecture (ZTA), biometric authentication, and blockchain-based identity verification.
Secure communication	Deploy TLS 1.3 with mutual authentication and software-defined security (SDS) for dynamic policy enforcement.
Intrusion detection and anomaly detection	Incorporate AI-driven intrusion detection systems (IDS) and behavioral analytics for real-time anomaly detection.
Data integrity and secure storage	Use distributed ledger technology (DLT) to ensure integrity across fog nodes.
Privacy-preserving computing	Implement differential privacy, federated learning, and secure multiparty computation (SMPC) to prevent unauthorized data exposure.

**Table 5:** Foundational pillars of IEEE 1934 [94].

Pillar	Description
Security	Ensures secure data transmission, storage, and processing across fog nodes using encryption, authentication, and blockchain-based security mechanisms.
Scalability	Supports the dynamic expansion of fog nodes, enabling the real-time provisioning of computational resources based on demand.
Openness	Provides open, standardized APIs and protocols for vendor-neutral integration across heterogeneous devices and platforms.
Autonomy	Supports decentralized decision-making, allowing fog nodes to process and act on data without requiring continuous cloud interaction.
Programmability	Enables software-defined control mechanisms, allowing flexible deployment and management of fog applications.
Ruggedization	Ensures the ability of fog systems to operate in harsh environments, including industrial IoT and mission-critical systems.
Hierarchy	Defines multitier fog node structures that optimize workload distribution between the cloud, fog, and edge layers.
Latency	Minimizes data transmission delays by processing data closer to the source, which is critical for real-time applications such as telepresence and autonomous vehicles.

**Table 6:** Functional layers in IEEE 1934 [94].

Layer	Functionality	Example Technologies
Application layer	Provides interfaces for users and services to interact with fog-based applications.	WebRTC, MQTT, Telepresence APIs
Orchestration layer	Manages distributed fog nodes, schedules tasks, and allocates resources dynamically.	AI-based resource schedulers, NFV, SDN Controllers
Data management layer	Handles data aggregation, filtering, and processing at different fog nodes.	Apache Kafka, TensorFlow Federated
Network layer	Ensures efficient data routing between fog, cloud, and edge devices.	5G/6G NR, time-sensitive networking (TSN)
Security layer	Implements identity authentication, access control, and encryption for secure communication.	Blockchain-based identity management, TLS, zero trust security
Infrastructure layer	Consists of fog nodes, edge devices, and cloud servers that execute computations.	GPUs, TPUs, MEC Servers, IoT Gateways

**Table 7:** Contributions of IEEE 1934 in 6G fog-telepresence evolution [94].

Aspect	IEEE 1934 Contributions
Low latency	Reduces latency in real-time telepresence applications by enabling localized processing at fog nodes.
Interoperability	Provides standardized APIs and protocols, ensuring seamless integration of different telepresence platforms.
Security and privacy	Implements secure access control and end-to-end encryption for telepresence data.
Scalability	Allows dynamic provisioning of computational resources for high-bandwidth holographic communication.
AI and edge intelligence	Supports federated learning and AI-driven decision-making at the edge for autonomous operations.

**Table 8:** Main components of 3GPP fog model.

Component	3GPP Terminology (Rel. 16–18)	Fog Computing Equivalent
Edge processing	Multiaccess edge computing	Fog nodes
Local data routing	Local breakout via UPF	Fog layer for traffic offloading
Low-latency services	uRLLC	Fog-enabled real-time applications
Distributed AI	Edge AI in 6G RAN	AI-driven fog processing
Interoperability	Network slicing and APIs for verticals	OpenFog standard (IEEE 1934)

**Table 9:** Comparison between fog models of IEEE, 3GPP, and ETSI.

Aspect	3GPP (5G/6G Fog-Enabled Features)	ETSI MEC	IEEE 1934 (OpenFog Standard)
Standardization	3GPP TS 23.501, 23.548 (Edge processing) [97]	ETSI MEC specifications (e.g., MEC 021) [98]	IEEE 1934: OpenFog reference model [94]
Scope	Focused on telecom networks	Edge computing within telecom networks	Distributed computing across various layers. It represents a generalized fog computing framework
Interoperability	Works within mobile networks	Works with 5G core, integrates NFV and SDN	Works across cloud-edge and provides general API-based interoperability
Use cases	5G/6G, IoT, C-V2X, XR	Low-latency applications (e.g., video analytics, V2X)	Ultrareliable low-latency applications, Industrial IoT, and smart cities

**Table 10:** Future research directions of fog computing.

Research Direction	Discussion
URLLC service	The research in this direction aims to develop methods that provide high reliability and low latency communications based on fog and mobile fog nodes.
SDN network	The research in this direction aims to manage and coordinate fog computing devices flexibly and efficiently.
Framework	The research in this direction aims to develop software frameworks that facilitate the easy and efficient deployment and management of fog computing applications. This also includes developing reliable platforms for fog nodes.
Algorithms	The research in this direction aims to develop algorithms to optimize resource utilization, data processing, and application allocation in a fog computing environment.
Devices	The research in this direction aims to develop energy-efficient fog computing devices that are high-performing and adaptable to various environments.

## Data Availability

No new data were created or analyzed in this study.
